# Renal Angiomyolipoma Causing Inferior Vena Cava Thrombus in a Young Girl With Tuberous Sclerosis

**DOI:** 10.7759/cureus.33244

**Published:** 2023-01-02

**Authors:** Omar Buksh, Ahmed Khogeer, Bader H Binyousef, Ayman Munshi, Abdulmonem M AlMutawa, Rana Alkhaibari, Zergham Zia, Islam Junaid

**Affiliations:** 1 Department of Urology, King Faisal Specialist Hospital and Research Centre, Jeddah, SAU; 2 Department of Pathology, King Faisal Specialist Hospital and Research Centre, Jeddah, SAU; 3 Department of Radiology, King Faisal Specialist Hospital and Research Centre, Jeddah, SAU

**Keywords:** female, early onset, angioembolization, aml, nephrectomy, inferior vena cava thrombectomy, case report, ivc thrombus, tuberous sclerosis, renal angiomyolipoma

## Abstract

Angiomyolipomas (AML), also known as hamartomas, are benign mesenchymal tumors of the kidneys which consist of vascular tissue, smooth muscles, and adipose tissue, with a higher prevalence in females than males. AML may be associated with tuberous sclerosis, and the growth of the mass may present as hematuria or flank pain. We present a case of a 14-year-old female patient who had a known case of tuberous sclerosis since early childhood. She has a history of numerous bilateral renal masses radiographically consistent with AML.

A special and unique entity of our case is the age of presentation which is 14 years and the presence of TSC. In contrast to our case, which was invading the right renal vein and IVC at a young age, AML is well known for its benign nature. According to a recent literature review, the youngest patient reported was 16 years old. Typically, non-complicated AMLs less than 4 cm in size are managed by annual radiological imaging which is preferably a CT scan, while larger AMLs of more than 4 cm that present with perinephric hemorrhages or intralesional aneurysms are treated by partial nephrectomy or selective angioembolization.

A radical nephrectomy and IVC thrombectomy are typically necessary due to the risks that the IVC thrombus carries as well as the AML itself and its unpredictable behavior. In cases like ours with the extension into the renal vein and IVC, the surgical approach is similar to the venous invasion of renal cell carcinomas.

## Introduction

Angiomyolipoma (AML), also known as hamartoma, is a benign mesenchymal tumor of the kidney consisting of vascular tissue, smooth muscles, and adipose tissue, with a higher prevalence in females [1,2]. AML may be associated with tuberous sclerosis and may present as hematuria or flank pain [3]. We present a rare case of AML invading the inferior vena cava (IVC) in a 14-year-old female with tuberous sclerosis.

## Case presentation

A 14-year-old female patient with a known case of tuberous sclerosis since early childhood and with a history of numerous bilateral renal masses radiographically consistent with AML presented to the emergency department. Her history was significant for elective surgery to place an angioembolization coil to control bleeding of a large left renal AML. Her chief complaint at presentation was moderate intermittent right flank pain that started suddenly two days prior and was not associated with abdominal distension or hematuria. Her vital signs and blood work, including hemoglobin, were normal. She was admitted for further workup and pain control.

**Figure 1:**
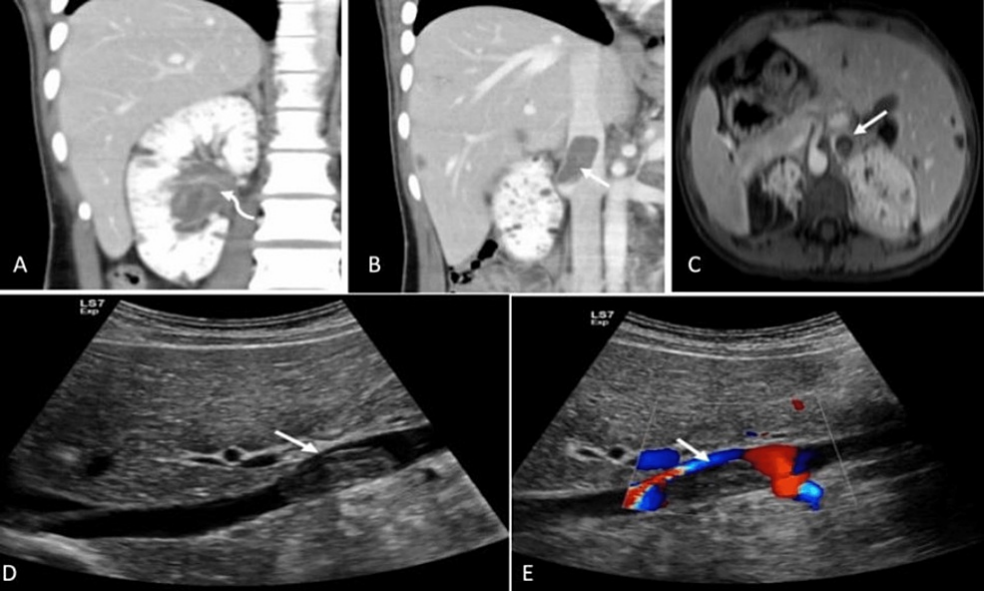
Contrast-enhanced CT scan of the abdomen and pelvis (a, b) Coronal portovenous contrast enhanced abdominal CT scan, (c) Axial T1-weighted VIBE sequence fat-saturated post contrast, (d) Grey scale and (e) color Doppler ultrasound images demonstrate a heterogeneous mass (curved arrow) in the right renal pelvis (a) with an apparent direct extension through the right renal vein into the IVC (b, c, d) with no internal vascularity (e) (arrow).

A contrast-enhanced CT scan of the abdomen and pelvis showed multiple small AMLs in the right kidney. A heterogeneous large mass in the right renal pelvis with hemorrhagic material appeared to extend directly through the right renal vein into the inferior vena cava (IVC). MRI confirmed the CT findings, showing a large filling defect in the right renal vein and its tributaries, extending into the IVC, approximately 3 cm in the craniocaudal dimension. The results were suggestive of a bland thrombus (Figure 1).

**Figure 2:**
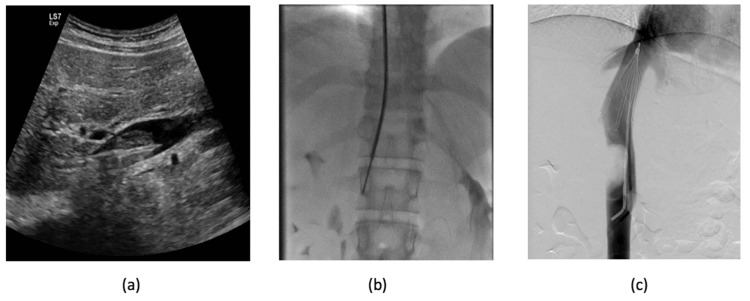
Intravenous biopsy of the thrombus Trans-jugular biopsy of the caval mass/thrombus under direct transabdominal ultrasound (a) and fluoroscopic guidance (b) with insertion of a suprarenal IVC filter (asterisk) (c).

Upon further evaluation, a mercaptoacetyltriglycine (MAG3) scan was done to assess split kidney function revealing a renal function of 34% and 66% in the left and right kidneys, respectively. Given the risk of pulmonary embolism with an IVC thrombus, a suprarenal IVC filter was inserted by the interventional radiologist. An intravenous biopsy of the thrombus was performed using a transjugular liver biopsy needle, and pathology confirmed the thrombus was an AML (Figure 2).

The decision for a right radical nephrectomy and IVC AML thrombectomy was made after counseling the parents and patient; consent was obtained after discussing the treatment plan, including the possible removal of the dominant functioning kidney. Retrieval of the IVC filter and embolization of the right renal artery was done under general anesthesia prior to the operation by the interventional radiologist. After transferring the patient to the surgical theater, a subcostal skin incision followed by a right radical nephrectomy and IVC thrombectomy were completed uneventfully. Multiple right renal AMLs were noted, as well as the thrombus which extended 2.5 cm through the renal vein into the IVC in a cranial direction. After the thrombectomy, a primary repair of the IVC was done, leaving more than 50% of the lumen intact with normal adrenals, and a single right renal vein and artery. On the left kidney, a lower-pole, large cyst was noticed. No blood transfusion was needed, with an estimated blood loss of 200 ml.

The patient had a smooth recovery and was discharged on her sixth postoperative day. Two weeks after discharge, she returned to our clinic. Her blood work was within normal levels, and the surgical site sutures were removed. On a subsequent outpatient follow-up at more than one year, the patient continued to be doing well, maintaining a creatinine level of 83 mmol/L.

**Figure 3:**
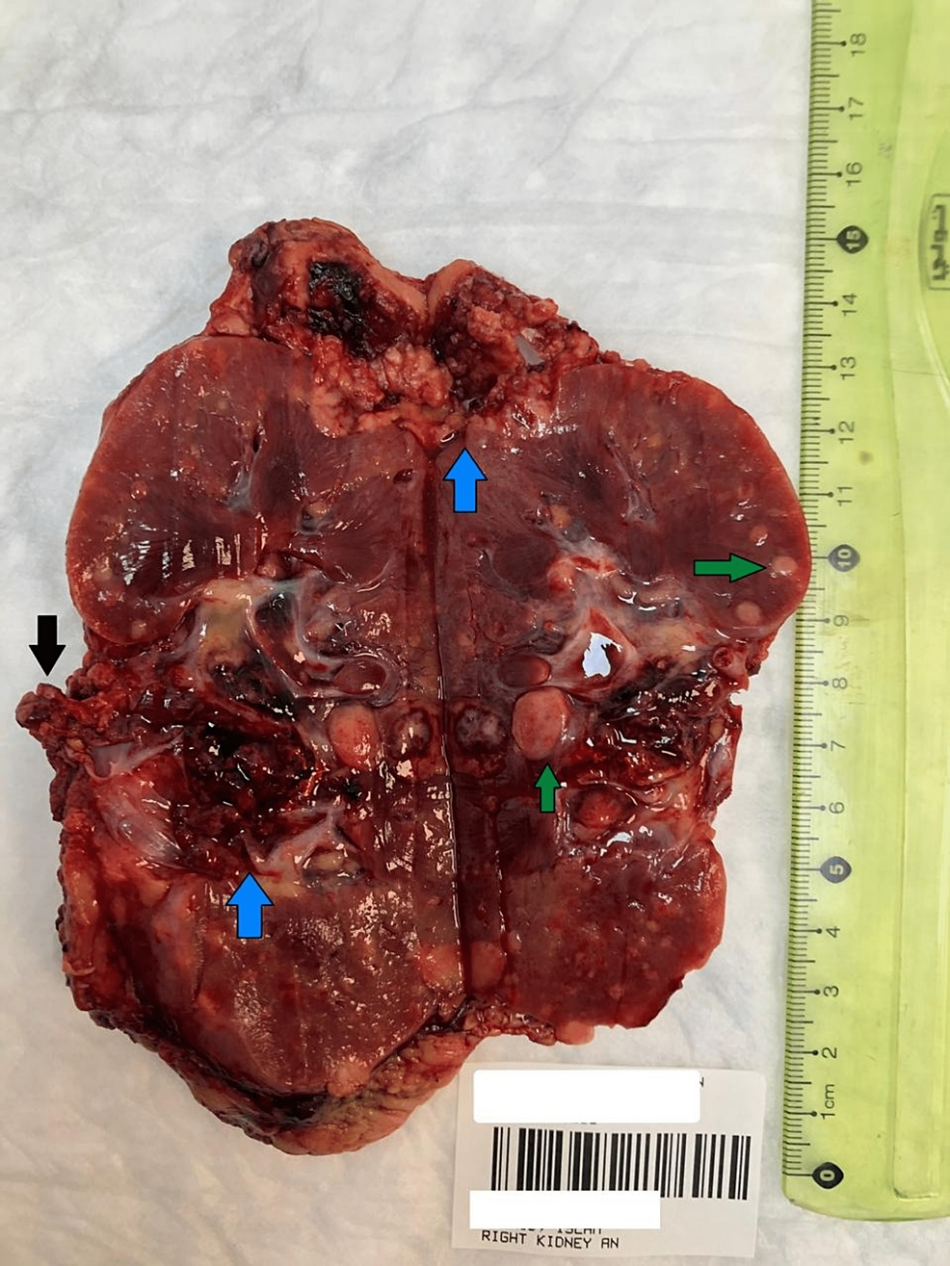
Right nephrectomy specimen Gross photograph of the right nephrectomy specimen, illustrating: two large tumor masses (blue arrows, one near the renal pelvis and the other is exophytic at the upper pole), a tumor embolus at the renal vein margin (black arrow), and large number of small discrete intraparenchymal tumor nodules (green arrows).

Pathologic examination of the right radical nephrectomy specimen revealed two relatively large intraparenchymal renal masses (Figure 3). The larger mass was 3.8 cm and was located near the renal pelvis. The smaller mass at the upper pole showed exophytic growth in perinephric fat. Both were hemorrhagic and light brown. A tumor embolus was noted at the renal vein margin. In addition, many small and discrete intraparenchymal nodules were scattered throughout the renal parenchyma. The nodules were firm and tan, ranging from 0.1 cm to 1.0 cm.

**Figure 4:**
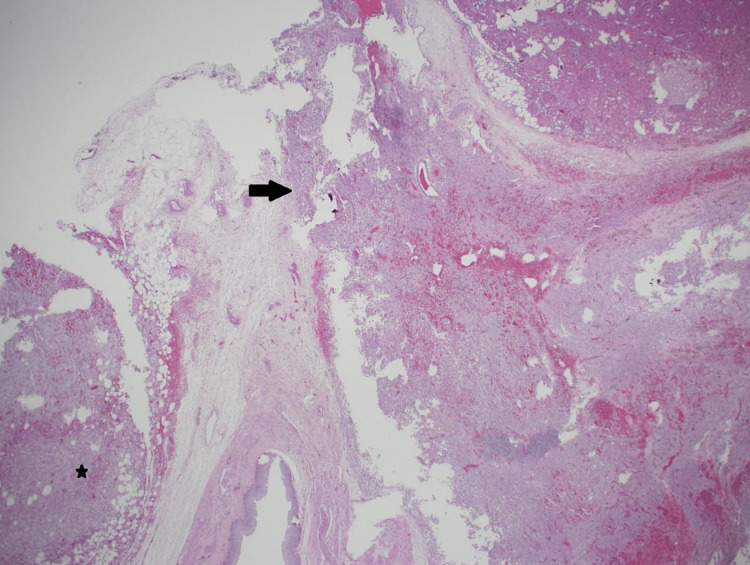
Microscopic photo Low magnification photomicrograph showing a tumor nodule (arrow) extending between renal cortical tissue (right upper) and pelvicalyceal system (lower central). Another tumor nodule is also seen (star). (H&E stain; original magnification 20x).

**Figure 5:**
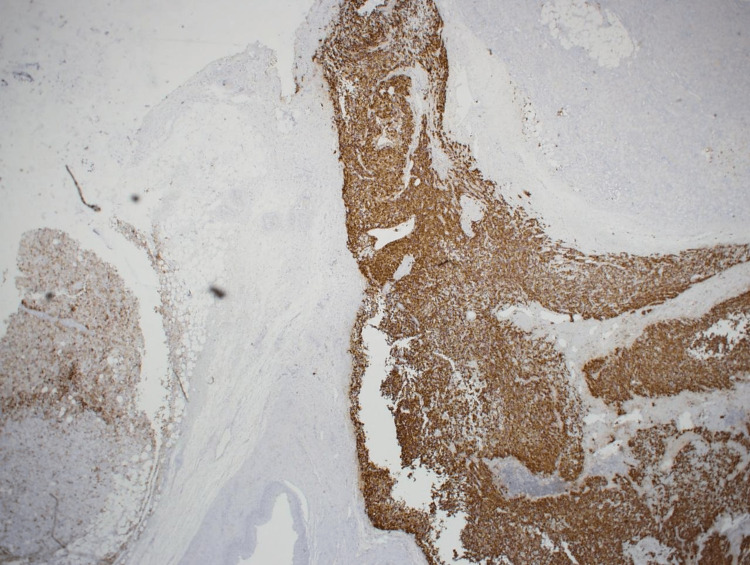
Microscopic photo HMB-45 immunohistochemical staining showing positive reaction in the tumor tissue noted in Figure 4. (original magnification 20x).

**Figure 6:**
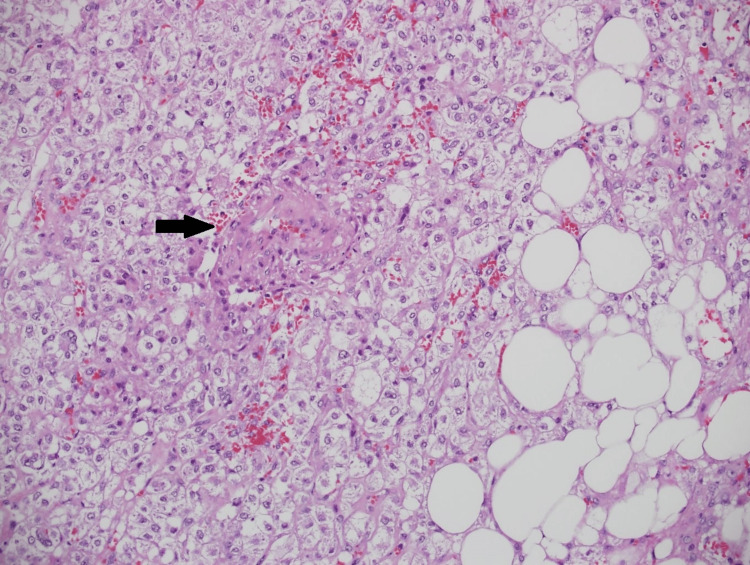
Microscopic photo High magnification photomicrograph illustrating various tumor components, including the smooth muscle cells (central and left), the fatty component (right), and a small vessel (arrow). (H&E stain; original magnification 200x).

Microscopic examination revealed two large masses exhibiting triphasic smooth muscle cells (epithelioid to slightly spindly, with granular to clear cytoplasm), mature adipose tissue, and occasional thick-walled vessels (Figures 4-5). An immunohistochemical study showed that the tumor cells were positive for HMB-45 and Mart-A (melan-A) markers but negative for Pax-8 (Figure 6). These pathologic features were consistent with AML.

**Figure 7:**
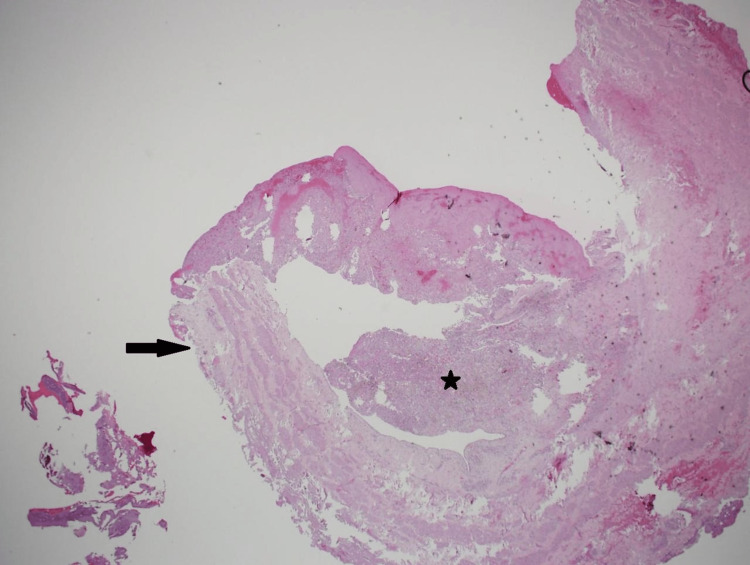
Microscopic photo Tumor thrombus (star) involving the renal vein (arrow) (H&E stain; original magnification 20x).

**Figure 8:**
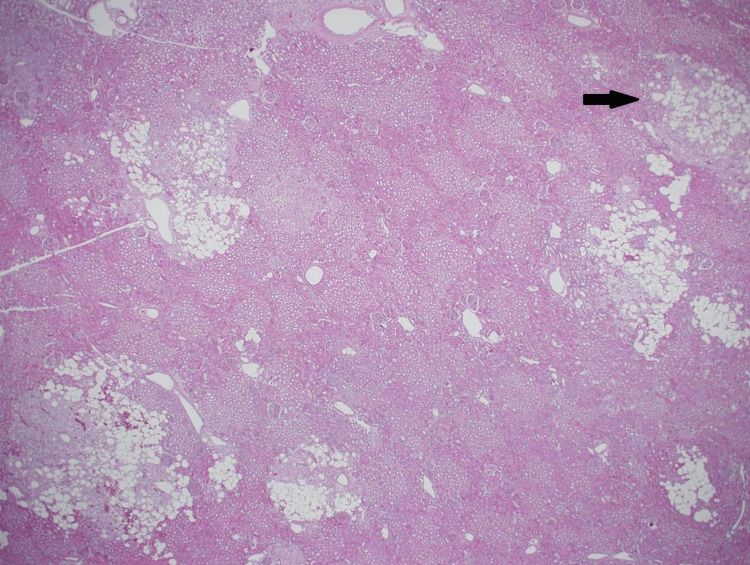
Microscopic photo The neoplastic cells forming multiple small discrete intraparenchymal nodules (microhamartomas) (arrow on one of them). (H&E stain; original magnification 20x).

The tumor involved the renal vein (in a thrombus-like extension) and extended to the renal vein margin, reaching focally to the perinephric fat margin (Figure 7). Small, discrete, intraparenchymal nodules noted grossly were microscopically formed of similar components as the two previously described larger masses, representing the so-called microhamartoma (Figure 8).

## Discussion

AMLs are the most common benign mesenchymal tumor of the kidneys [1,2]. About 80% of cases are found incidentally on radiological studies with no known association of this renal tumor to other diseases. About 25% of patients with tuberous sclerosis complex also have lymphangiomyomatosis, which also may be associated with AML. This type of renal tumor is generally solitary but can occur with tuberous sclerosis complex. AMLs can be bilateral and multifocal [3].

The first AML associated with IVC invasion was reported in 1982 by Kutcher et al. Most cases occur in patients over 40 years old, with the youngest being reported as 16 years old [2]. To the best of our knowledge, 77 cases of AML include involvement with the IVC, and approximately 75% of all cases of aggressive AML are sporadic and isolated [4,5]. Early detection of AML mainly relies on radiological studies. Ultrasound is an effective screening option, with CT scans having the greatest diagnostic value [6]. AMLs are rich in adipose tissue and appear on CT scans in a hypodense pattern of about 20 house field units. Approximately 5% lack fat content and cannot be differentiated from renal cell carcinoma. Thus, these variants of AML are difficult to diagnose via CT. Various studies propose that MRI may improve diagnostic accuracy with the use of fat saturation techniques [7].

Typically, uncomplicated AMLs smaller than 4 cm are managed by annual radiological imaging, preferably CT scans, and larger ones accompanied by perinephric hemorrhages or intralesional aneurysms can be treated by partial nephrectomy or selective angioembolization [8]. In cases with extension into the renal vein and IVC, the surgical approach is similar to that for venous invasion of renal cell carcinoma. A radical nephrectomy and IVC thrombectomy are usually required, though this approach carries the same risks as an IVC thrombus along with the unpredictability of the AML itself.

## Conclusions

AML is a benign renal tumor that may present as part of a spectrum of tuberous sclerosis complexes. AMLs are usually treated by angioembolization, as indicated. However, on rare occasions, they may behave aggressively and invade the IVC, necessitating more surgical management and complete removal of the kidney. This case highlights the importance of accurately diagnosing AML in pediatric patients to ensure proper treatment.
